# Cuadragésimo aniversario de la revista Biomédica

**Published:** 2021-12-15

**Authors:** Luis Alberto Gómez-Grosso, José Moreno-Montoya

**Affiliations:** 1 Editor en jefe, revista Biomédica, Instituto Nacional de Salud Facultad de Medicina, Universidad Nacional de Colombia Universidad Nacional de Colombia Instituto Nacional de Salud Facultad de Medicina Universidad Nacional de Colombia Colombia; 2 Editor Asociado, revista Biomédica, Instituto Nacional de Salud Fundación Santa Fe de Bogotá Facultad de Medicina, Universidad Nacional de Colombia Universidad Nacional de Colombia Instituto Nacional de Salud Fundación Santa Fe de Bogotá Facultad de Medicina Universidad Nacional de Colombia Colombia

En este 2021, la revista *Biomédica* celebró su cuadragésimo aniversario. El primer volumen, editado por el doctor Miguel Guzmán (Q.E.P.D.), fundador y primer editor de la revista, se publicó en 1981 con cuatro números, 30 artículos y 271 páginas. El volumen actual de la revista, el 41, del cual somos responsables 11 editores, consta de siete números (cuatro números regulares y tres suplementos) con 102 artículos y más de 680 páginas (figura 1). Este crecimiento sostenido es reflejo de un denodado esfuerzo editorial, y ratifica a *Biomédica* como la revista científica colombiana más productiva y la que publica mayor número de trabajos de científicos latinoamericanos del campo de la salud y la biomedicina, con una cantidad de manuscritos por año que supera entre dos y cinco veces la de otras revistas nacionales en este campo.

**Figura 1:**
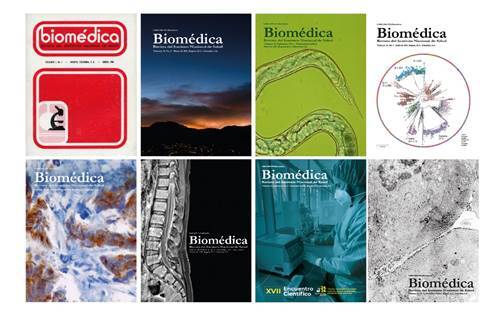
Portada del número 1 del volumen 1 de 1981 y de los números 1, 2, 3 y 4 y de los suplementos 1, 2 y 3 del volumen 41 de 2021

Es innegable que la revista ha aumentado su productividad, pero son muchos otros los motivos para celebrar (figura 2). En el 2002, *Biomédica* fue incluida en el *Index Medicus*; en el 2007, en el *Science Citation Index* y en el 2021, en *PubMedCentral*^1^^-^^3^. Además, *Biomédica* es una de las 23 revistas seleccionadas a nivel internacional por el *Journal Citation Reports/Web of Science* en la categoría de medicina tropical y es hoy la única de ellas que publica artículos en español o en inglés.

**Figura 2:**
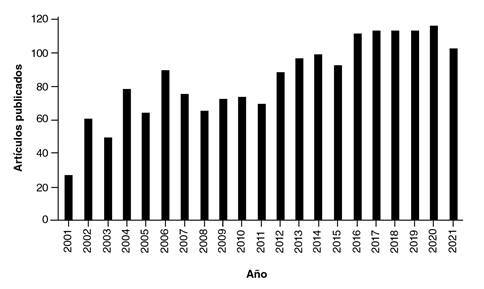
Número de artículos publicados en Biomédica, 2001-2021

*Biomédica* fue seleccionada para participar en el Grupo de Influencia de la Red Iberoamericana de Innovación y Conocimiento Científico, REDIB, un proyecto del Consejo de Investigaciones Científicas de España y *Universia*, la red de universidades más importante de Iberoamérica, constituida por 1.341 instituciones de 23 países que agrupan a más de 19,2 millones de estudiantes y profesores. REDIB es una plataforma de agregación de contenidos científicos en formato electrónico, cuyo fin es promover la innovación tecnológica de las herramientas de producción editorial y facilitar el acceso y la difusión de la producción científica de los países iberoamericanos en el idioma que les sea propio. Los destinatarios de esta información son la comunidad académica y la sociedad en general, así como los responsables, gestores y analistas de políticas de ciencia. Con su participación en REDIB, *Biomédica* se posiciona como protagonista en el desarrollo científico de la región, a la vez que favorece la promoción de los investigadores.

Durante estos 40 años, y pese a no ser la revista más antigua del país en el área de la biomedicina, la salud pública y la medicina tropical ^2^, *Biomédica* ha sido ampliamente reconocida como la principal revista científica de Colombia en estos campos y como una de las principales y más influyentes de la región iberoamericana ^4^. Estos logros son consecuencia del inagotable compromiso por mantener los más altos estándares de calidad en los procesos de evaluación científica y editorial de los manuscritos ^5^^,^^6^.

Como fruto de este esfuerzo, la revista también hace parte de SciELO Colombia (*Scientific Electronic Library Online*) desde el 2006, y aparece reseñada en el índice de la *Literatura Latinoamericana en Ciencias de la Salud* (LILACS), en la *Red de Revistas Científicas de América Latina*, el Caribe, España y Portugal (RedAlyC), en el *Índice Mexicano de Revistas Biomédicas Latinoamericanas* (Imbiomed), en Scopus de Elsevier B.V., en el *Sistema de Información Bibliográfica Regional Andina* (SIBRA), en *CAB Abstracts*, y en la *Review of Medical and Veterinary Entomology*; además forma parte del *Índice Nacional de Publicaciones Seriadas* Científicas y *Tecnológicas Colombianas de Colciencias* (Publindex) y del *Índice Latinoamericano de Revistas Científicas y Tecnológicas* (LATINDEX).

Desde el 2012, la revista se suscribió a la agencia internacional *CrossRef*, encargada de la asignación de los *Digital Object Identifiers* (DOI). Por tal razón, a partir del volumen 32 todos los artículos publicados en Biomédica cuentan con el código DOI que los identifica como piezas únicas de contenido electrónico y establece un vínculo estable para su localización en la web ^7^^,^^8^.

Desde el 2013, *Biomédica* viene publicando de manera gratuita la totalidad de los artículos en formato electrónico en su portal institucional: www. revistabiomedica.org, con más de medio millón de visitas de más de 170 países cada año, la mayoría correspondiente a nuevos usuarios (80 %) ^9^. En ese año, también se acogió a la Declaración de San Francisco, una serie de recomendaciones sobre la evaluación de la investigación científica redactada por un grupo de directores y editores de las revistas académicas más prestigiosas del mundo ^10^.

Con el fin de motivar la lectura e intensificar la difusión de los artículos publicados en cada edición de Biomédica, a partir del 2014 se incorporaron diversas estrategias de mercadeo digital en las principales redes sociales y académicas del mundo, así como el envío masivo de boletines con información de interés para los lectores. Periódicamente, en las redes se publican resúmenes con las ideas más importantes de los artículos, acompañados de imágenes alusivas al tema y un vínculo al texto completo. Además, se han usado las denominadas etiquetas, que permiten que los contenidos de *Biomédica* puedan hacer parte de los temas de conversación de diferentes usuarios conectados a la red. Desde el 2015, en la página web de Biomédica, se puede descargar gratuitamente una aplicación para dispositivos móviles que permiten la descarga y lectura de artículos completos en celulares y tabletas con las plataformas móviles iOS y Android ^11^^,^^12^.

Por otra parte, en cumplimiento del plan de mejoramiento continuo de *Biomédica*, recientemente se revisó la política editorial y las instrucciones a los autores, y se mantiene la participación en eventos nacionales e internacionales sobre temas de gestión editorial de actualidad, sistemas digitales para la visibilidad, citación e identidad de revistas científicas, malas prácticas en la publicación científica (revistas "depredadoras"), recomendaciones para la escritura de artículos científicos y el uso e implementación de herramientas tecnológicas que contribuyen a mejorar la calidad, la visibilidad y el acceso a los contenidos de la revista.

Velando por la transparencia y originalidad de las publicaciones científicas, y en aras de la integridad de la información publicada por *Biomédica*, a partir del 2018 el Comité Editorial implementó el uso de la herramienta *iThenticate* contra el plagio en los procesos de revisión editorial de los manuscritos recibidos para publicación en la revista.

Debe señalarse también que, entre 1981 y 2006, *Biomédica* se publicó únicamente en medio impreso, que a partir del 2007 y hasta diciembre del 2017 apareció en medio digital e impresa, y desde el 2018 se viene publicando exclusivamente en medio digital en consonancia con las tendencias actuales en el campo de las publicaciones científicas a nivel global. En el 2021, *Biomédica* se unió a la *Initiative for Open Citations* (https://i4oc.org), un ámbito de colaboración entre editores, investigadores y otras partes interesadas en promover la disponibilidad ilimitada de datos de citas académicas. Por medio de esta iniciativa, no solamente se emiten los códigos DOI y los sellos *Crossmark*, sino que se cargan las referencias de cada uno de los artículos de *Biomédica* en el sistema *CrossRef* para su posterior consulta por parte de lectores e investigadores.

Para conmemorar este aniversario, el equipo editorial de *Biomédica* planificó un concurso de fotografía científica en que se seleccionaron las ganadoras en seis categorías, las cuales serán portada de los números regulares y los suplementos temáticos del volumen 42, así como la realización del evento “*Biomédica en PubMed Central*”, que puede verse en https://www.youtube.com/watch?v=8vN0WjneSHM, en el cual se presentó una breve historia de la indización y la producción de las publicaciones científicas, así como de los procesos editoriales y los principales repositorios. Se destacó la importancia de haber sido aceptados en *PubMed Central*® (PMC), el más prestigioso repositorio central y digital de artículos de revistas biomédicas y de ciencias de la vida en texto completo, el cual hace parte de la *National Library of Medicine* de los *National Institutes of Health* (NLM/ NIH) de los Estados Unidos ^13^. En el evento se explicó, asimismo, lo que es PubMed Central, lo que representa para la comunidad científica estar en dicha base de datos y cuáles fueron algunos de los factores de éxito que nos permitieron este logro. Se presentó, además, una breve reseña histórica de la revista desde su fundación en 1981 hasta el 2021.

Los esfuerzos y el compromiso no cesan. El Comité Editorial continúa comprometido en mantener la calidad científica y editorial de la revista y en aumentar su visibilidad e importancia, no solo en el campo de la biomedicina y la medicina tropical, sino en todas las disciplinas en las que la salud pública constituye uno de los temas.

Por todo ello, prevalece en nuestro equipo la confianza de que nos esperan muchos más años en este camino de apoyo a la divulgación y la cooperación científica. La publicación ininterrumpida de la revista en estos 40 años refleja el incansable interés y el compromiso de los editores y los revisores expertos, de tantos autores influyentes y, por supuesto, del equipo editorial. Agradecemos profundamente el aporte de todos ellos, así como el del Instituto Nacional de Salud por garantizar la autonomía y la independencia de la revista y por la gestión y promoción que le han permitido mantenerse durante estas cuatro décadas.

Estas líneas no representan solamente un reconocimiento a los logros y labores desarrolladas, sino una invitación para que la comunidad científica continúe contribuyendo a ellas con la calidad y el rigor científico y editorial que caracterizan a *Biomédica*. Felicitaciones a todos los que hacen parte de la revista.

Un nuevo viaje de 40 años acaba de empezar...

## References

[1] Gómez-Grosso LA (2020). Biomédica en PubMed Central®. Biomédica.

[2] Comité Editorial (2012). Biomédica: diez años en el Index Medicus y cinco en el Science Citation Index. Biomédica.

[3] Science Citation Report Sciences Edition (2021). Clarivate Analytics.

[4] International Committee of Medical Journal Editors Uniform requirements for manuscripts submitted to biomedical journals.

[5] Aguinis H, Villamor I, Lazzarini SG, Vassolo RS, Amorós JE, Allen DG (2020). Conducting management research in Latin America: why and what’s in it for you. Journal of Management.

[6] Suárez-Tamayo M, Collazo-Reyes F, Pérez-Angón MÁ (2018). Emerging roles of regional journals in the accreditation of knowledge in tropical medicine: Biomédica and Memorias do Instituto Oswaldo Cruz, 2007-2015. Scientometrics.

[7] Infobliblio Información bibliográfica. DOI: un código esencial para citas bibliográficas y búsquedas científicas.

[8] CrossRef DOI®.

[9] Google.com Features - Google Analytics.

[10] Schmid SL (2017). Five years post-DORA: promoting best practices for research assessment. Mol Biol Cell.

[11] Biomédica on the App Store https://apps.apple.com/us/app/biom%C3%A9dica/id107392956911.

[12] Biomédica en Google Play https://play.google.com/store/apps/details?id=com.biteca.biomedica&hl=esCO.

[13] National Institutes of Health, US National Library of Medicine About the National Library of Medicine.

